# Orientation-specific surround suppression in the primary visual cortex varies as a function of autistic tendency

**DOI:** 10.3389/fnhum.2014.01017

**Published:** 2015-01-06

**Authors:** Anastasia V. Flevaris, Scott O. Murray

**Affiliations:** Department of Psychology, University of WashingtonSeattle, WA, USA

**Keywords:** surround suppression, autism quotient, perception, contextual modulation, fMRI

## Abstract

Individuals with autism spectrum disorder (ASD) exhibit superior performance on tasks that rely on local details in an image, and they exhibit deficits in tasks that require integration of local elements into a unified whole. These perceptual abnormalities have been proposed to underlie many of the characteristic features of ASD, but the underlying neural mechanisms are poorly understood. Here, we investigated the degree to which orientation-specific surround suppression, a well-known form of contextual modulation in visual cortex, is associated with autistic tendency in neurotypical (NT) individuals. Surround suppression refers to the phenomenon that the response to a stimulus in the receptive field of a neuron is suppressed when it is surrounded by stimuli just outside the receptive field. The suppression is greatest when the center and surrounding stimuli share perceptual features such as orientation. Surround suppression underlies a number of fundamental perceptual processes that are known to be atypical in individuals with ASD, including perceptual grouping and perceptual pop-out. However, whether surround suppression in the primary visual cortex (V1) is related to autistic traits has not been directly tested before. We used fMRI to measure the neural response to a center Gabor when it was surrounded by Gabors having the same or orthogonal orientation, and calculated a suppression index (SI) for each participant that denoted the magnitude of suppression in the same vs. orthogonal conditions. SI was positively correlated with degree of autistic tendency in each individual, as measured by the Autism Quotient (AQ) scale, a questionnaire designed to assess autistic traits in the general population. Age also correlated with SI and with autistic tendency in our sample, but did not account for the correlation between SI and autistic tendency. These results suggest a reduction in orientation-specific surround suppression in V1 with increasing autistic tendency.

## Introduction

There is abundant evidence for atypical perceptual processing in individuals with Autism Spectrum Disorder (ASD). Indeed, sensory processing differences have been characteristic to clinical descriptions of ASD since the original reports of the disorder (Kanner, 1943; Asperger, 1944), and have recently been added as diagnostic criteria in the DSM-5 (American Psychiatric Association, 2013). In the visual domain, individuals with ASD exhibit enhanced “local” processing (i.e., perception of detail) and diminished “global” processing (i.e., integrating perceptual features into a unified whole) compared to the neurotypical (NT) population (e.g., Dakin and Frith, 2005; Simmons et al., 2009). For example, individuals with ASD are better at copying impossible figures (Mottron et al., 1999), possibly resulting from an impaired ability to see, and so be distracted by, impossibilities in the overall structure (Brosnan et al., 2004). A similar lack of “distraction by context” has been used to explain why individuals with ASD, and NT individuals with autistic tendencies, are less susceptible to some visual illusions (e.g., Happé, 1996; Walter et al., 2009; Chouinard et al., 2013). Individuals with ASD also excel in other paradigms in which ignoring context aids performance, such as feature-conjunction search tasks (Plaisted et al., 1998; O’Riordan and Plaisted, 2001; O’Riordan et al., 2001; O’Riordan, 2004; Kemner et al., 2008), in which individuals with ASD are better at finding targets embedded in distractors that share features with the target.

In addition to enhanced local processing, studies have also demonstrated deficits in global visual processing in individuals with ASD, particularly in tasks that require linking together individual elements to form coherent shapes, surfaces, and objects (e.g., Rinehart et al., 2000; Blake et al., 2003; Behrmann et al., 2006; Del Viva et al., 2006; Kemner et al., 2007; Wang et al., 2007). However, the evidence for a global processing deficit is mixed. For example, using hierarchical letter displays in which a series of smaller “local” letters are arranged to form a larger “global” letter (Navon, 1977), some studies have found that individuals with ASD are impaired at reporting the global letter relative to NT controls (e.g., Rinehart et al., 2000; Behrmann et al., 2006; Wang et al., 2007), whereas other studies found no difference in performance between the two groups (Plaisted et al., 1999; Mottron et al., 2003; Iarocci et al., 2006; Scherf et al., 2008; Hayward et al., 2012). A recent study (Koldewyn et al., 2013) offered an intermediate perspective that individuals with ASD may have a “disinclination”, but not a disability, in processing global information. Global processing deficits have also been shown using Gestalt grouping paradigms (e.g., Brosnan et al., 2004), but other studies examining grouping and contour integration in ASD have not found differences between individuals with ASD and NT controls (Blake et al., 2003; Del Viva et al., 2006; Kemner et al., 2007; Farran and Brosnan, 2011). Studies examining coherent motion discrimination have found individuals with ASD to require about 10 percent more coherent motion to correctly report the direction of overall motion in a set of moving dots (Milne et al., 2002; Tsermentseli et al., 2008), suggesting a general impairment in spatio-temporal integration. Studies have also found that individuals with ASD have a deficit in zooming-out visual attention, which could also impair spatio-temporal integration (Ronconi et al., 2012, 2013).

The neural mechanisms underlying the perceptual processing differences exhibited by individuals with ASD are currently unknown. Comprehensive behavioral measurements of contrast sensitivity across a broad range of spatial frequencies revealed no differences between individuals with ASD and NT controls (Koh et al., 2010), suggesting that there are not fundamental changes in neural response properties in early visual areas. There may be differences in lateral connectivity in early visual cortex, supported by behavioral studies using detection thresholds of targets with lateral flankers (Kéïta et al., 2011), and enhanced cortical representation of peripheral visual space (Frey et al., 2013). A meta-analysis of fMRI studies that used visual stimuli (e.g., faces, words, etc.), found general increases in the fMRI response in occipital (visual) cortex compared to controls (Samson et al., 2012), consistent with a reduction in lateral connectivity in visual cortex. These findings are also consistent with an emerging neurobiological theory of ASD, which suggests that ASD results from an increase in the ratio of cortical excitation to inhibition (“E/I balance”), which could arise from disproportionally high levels of glutamatergic excitation or disproportionately low levels of GABAergic inhibition (Rubenstein and Merzenich, 2003; Markram and Markram, 2010; Rubenstein, 2010; Vattikuti and Chow, 2010).

A recent study examining motion discrimination thresholds for stimuli varying in size also found evidence that there may be reduced inhibition with large motion stimuli in individuals with ASD, supporting a possible E/I imbalance (Foss-Feig et al., 2013). For NT individuals, motion direction becomes more difficult to perceive as stimuli size increases (Tadin et al., 2003), which is believed to reflect surround suppression in visual area MT (e.g., Tadin et al., 2003; Churan et al., 2008). Surround suppression is a well-known form of contextual modulation in visual cortex in which the response to a stimulus in the receptive field of a neuron is reduced when it is surrounded by stimuli just outside the receptive field (e.g., Blakemore and Tobin, 1972; Allman et al., 1985; Cavanaugh et al., 2002; Zenger-Landolt and Heeger, 2003). In the case of motion stimuli, it is assumed that perceiving the motion direction of a drifting Gabor becomes difficult with increasing stimulus size in NT individuals because in the case of a larger Gabor, the portion outside the receptive field suppresses the perception of the center portion (Tadin et al., 2003). Foss-Feig et al. (2013) showed that individuals with ASD did not exhibit an increase in motion discrimination thresholds relative to NT individuals for larger stimulus sizes, at least for low contrast stimuli, suggesting a reduction in surround suppression in the visual cortex of individuals with ASD. In addition to motion stimuli, surround suppression has been demonstrated using static displays, in which a central, static Gabor is surrounded by a Gabor of the same vs. orthogonal orientation (e.g., Blakemore and Tobin, 1972; Polat and Sagi, 1993, 1994; Cavanaugh et al., 2002; Mazer et al., 2002; Serrano-Pedraza et al., 2012). Orientation-specific surround suppression has been commonly reported, in which suppression is greatest when the surround and center gratings have the same orientation, and suppression decreases as the surround orientation deviates from the center orientation. Suppression is measured either psychophysically, as an increase in contrast discrimination threshold of the center grating as a function of its surround, or neurophysiologically, as a reduction in the neural response to the center grating. Similar to the findings with motion stimuli, a recent psychophysical study investigated contrast discrimination of a central sinusoidal grating flanked by parallel vs. orthogonal surrounds in NT individuals of varying autistic tendencies, and found a reduction in orientation-specific surround suppression in individuals with higher autistic tendencies relative to individuals with lower autistic tendencies (Van Heer and Crewther, 2012). However, studies to date have not investigated suppression in the neural response in individuals of varying autistic tendency.

Growing evidence suggests that the perceptual abnormalities exhibited by individuals with ASD are also present in NT individuals with high autistic tendencies, as measured by the “Autism Quotient” (AQ) scale (Baron-Cohen et al., 2001). The AQ is a questionnaire that was designed to measure the degree of autistic tendency in adults with typical intelligence, applicable to individuals with high-functioning autism or Asperger’s disorder, as well as NT individuals without an ASD diagnosis. Similar to studies of individuals with ASD, studies investigating perceptual processing as a function of AQ have shown that individuals who score high on the AQ (i.e., who have high autistic tendencies) exhibit increased sensory sensitivities (Robertson and Simmons, 2013) as well as enhanced local and reduced global perception across a range of tasks (e.g., Bayliss and Tipper, 2005; Sutherland and Crewther, 2010; Crewther, 2011; Kasai and Murohashi, 2013; Crewther and Crewther, 2014). For example, when manipulating the saliency of global and local letters by either blurring the display (i.e., enhancing global saliency) or randomly coloring the individual local letters (i.e., enhancing local saliency), Sutherland and Crewther (2010) showed that individuals with higher AQ scores were less able to ignore the salient local elements relative to individuals with lower AQ scores. To investigate the neural basis for enhanced local and/or reduced global perception that is associated with the autistic trait, in the present study we used fMRI to measure orientation-specific surround suppression in NT individuals as a function of AQ.

## Methods

### Participants

Fourteen students (ten women) from the University of Washington (ages 19–31) participated in the experiment for monetary compensation. All gave written informed consent as approved by the University of Washington Institutional Review Board. Three participants were excluded from the analyses due to excessive head motion in the scanner (>1 mm), leaving a total of eleven participants (seven women).

### Autism quotient (AQ) scale

All participants filled out the AQ questionnaire (Baron-Cohen et al., 2001), a scale of 50 questions related to social and environmental behavioral patterns. The AQ scale was scored according to Baron-Cohen et al. (2001) and AQ score was correlated with fMRI measures in each participant.

### Stimuli

During testing participants maintained fixation on a central black fixation cross that subtended 0.4° and remained on the screen throughout the experiment. Six small black circles subtending 0.3° surrounded the fixation cross. The two center fixation circles were located 0.2° to the left and right of the cross, and the remaining fixation circles were 0.2° above and below the center circle on each side. These small black circles signified the location of six Gabor patches that each subtended 3.8° and that were presented 3.8° to the left and right of the fixation cross, three on each side. The center-to-center distance of each center Gabor to the upper and lower flanking Gabors was 3.8°. The Gabor patches were generated using the MATLAB Psychtoolbox (Brainard, 1997), and had a SF of 2 cycles per degree (cpd), 75% contrast, and were Gaussian-windowed with 0.7° standard deviation. The central Gabors had vertical orientation, and the flanking Gabors had either vertical or horizontal orientation, yielding collinear and orthogonal conditions, respectively. We kept the central Gabors at a fixed orientation, and only varied the surrounding flankers, in order to compare the BOLD response to physically identical stimuli. Any bias in orientation would thus not influence the BOLD response to the central Gabor, since its orientation remained constant. During each block one of the six small circles was colored white, to signify the location of the Gabor patch that was to be attended during that block.

### Procedure

Trial timing was controlled by Presentation (Neurobehavioral Systems, Albany, CA. Participants were each run on two localizer scans and four stimulus scans. A localizer scan (252 s) consisted of three stimulus conditions presented in alternating 12 s blocks: (1) fixation (“F”), (2) stimulus block with checkerboards at the location of the center Gabors (“C”), and (3) stimulus block with checkerboards at the location of the flanking Gabors (“F”). The fixation condition was inserted between each stimulus condition and the scan began and ended with the fixation condition (F—C—F—U—F …F). The checkerboards were Gaussian-windowed with 0.7° standard deviation and counter-phase flickered at 10 Hz.

**Figure 1:**
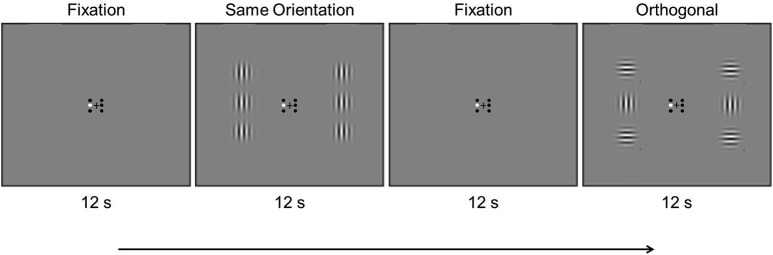
**Example timing of an experimental scan showing the first four blocks**. Stimulus conditions were presented in 12 s alternating blocks, in which the Gabors counter-phase flickered at 0.5 Hz (see text).

A stimulus scan (300 s) consisted of three stimulus conditions presented in alternating 12 s blocks (Figure 1): (1) fixation (“F”), (2) stimulus block with center and flanking Gabors at the same orientation (“S”), and (3) stimulus block with center and flanking Gabors at an orthogonal orientation (“O”). Akin to the localizer scan, the fixation condition was inserted between each stimulus condition and the scan began and ended with the fixation condition (F—S—F—O—F …F). The Gabors counter-phase flickered at 0.5 Hz to prevent adaptation effects. Contrast changes occurred twice in random order on each Gabor during each 12 s block. A contrast change consisted of either a decrease from 75% to 50%, or an increase to 100%. For a given contrast change, whether the contrast increased or decreased was random. Participants were instructed to attend to one Gabor location in each stimulus scan and look for contrast changes at the attended location, indicating via button press if the contrast increased or decreased during such changes. Participants attended the center left and center right locations in separate scans in counterbalanced order, and each location was attended twice in an experimental session. Participants were also run on four stimulus scans not reported here, in which they attended to the flanking Gabors as part of a separate study examining the effect of attention on surround suppression. The order of conditions was counterbalanced across participants, and the corresponding small fixation circle was colored white to remind participants which Gabor they should attend in each scan.

### fMRI acquisition and analysis

Functional MRI data were acquired using a Philips Achieva 3T scanner using a 32-channel head coil and an echo-planar imaging sequence (repetition time, 2 s; flip angle, 70°; 31 axial slices of 3.5 mm thickness (no gap) and 3.44 × 3.44 mm resolution, field of view, 220 mm). Each scanning session began with a T1-weighted structural scan with 1 × 1 × 1 mm resolution used for visualization of retinotopic visual areas. Visual cortical area V1 was localized using standard retinotopic mapping techniques using BrainVoyager QX.

**Figure 2:**
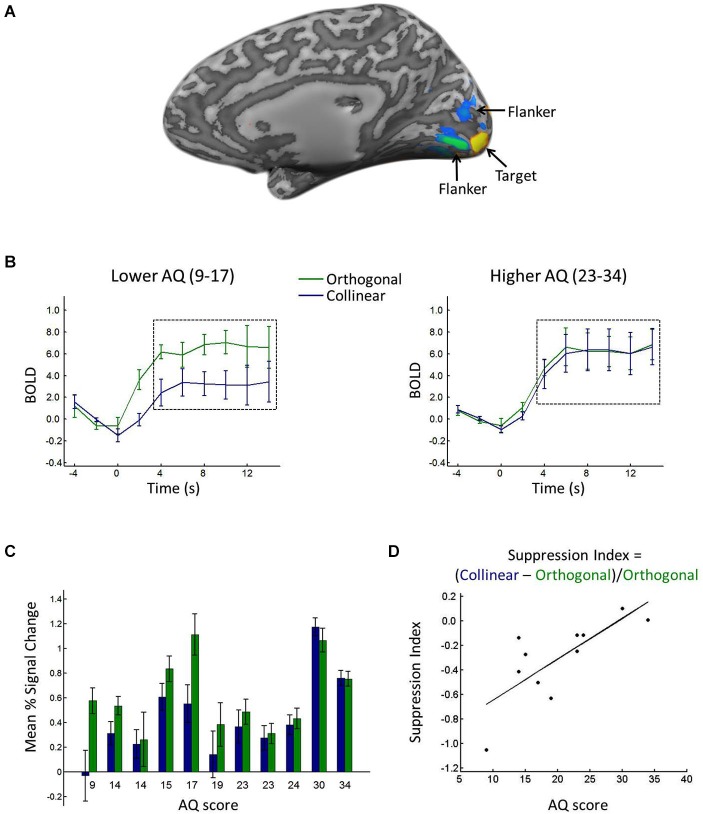
**(A)** Depiction of ROI. Voxels with significantly greater response to the target (yellow) and flanker (blue/green) locations during the localizer scan. **(B)** BOLD response in the target ROI when the target was surrounded by collinear (blue) vs. orthogonal (green) flankers, shown for lower (left) vs. higher (right) AQ participants, calculated from a median split (excluding the median score). Black dashed boxes show the time points averaged to yield the mean percent signal change (see text for details). Error bars show between-participant standard error. **(C)** Average mean percent signal change in the target ROI when the target was surrounded by collinear (blue) vs. orthogonal (green) flankers, shown separately for each participant in rank order of AQ score. Error bars show standard error across blocks. **(D)** Suppression index for each participant plotted against his/her AQ score.

Regions of interest (ROIs) in the left and right primary visual cortex (V1) were defined using the localizer scan; voxels with a significantly larger response to the center location relative to the flanker location in V1 were included in further analyses (Figure 2A). Using a false discovery rate (FDR) criterion of *q* < 0.05, we selected up to 37 active contiguous voxels, on average selecting 18 voxels for each participant. Time courses for each of the stimulus scans were extracted and averaged across voxels within each ROI (Figure 2B). The signal intensity in each condition was time-locked to the onset of the stimuli and averaged, from 2 s before the onset to 10 s after the onset. The 2 s before stimulus onset served as a baseline, and the percent signal change relative to the baseline was calculated and used as the measure of mean percent signal change in each condition.

### Comparison with AQ

Mean percent signal change in the left and right V1 ROI were averaged to generate one value for the collinear and orthogonal conditions, respectively. A suppression index (SI) was calculated for each participant by subtracting the mean percent signal change in the same orientation condition minus the orthogonal condition, and dividing by the mean percent signal change in the orthogonal condition (i.e., (S−O)/O). More negative values thus indicated greater surround suppression. The SI was then correlated with AQ score.

## Results

### AQ scores

Autism Quotient scores ranged from 9 to 34, and the median score was 19. Table 1 shows the AQ scores, gender and ages of the participants.

**Table 1:** **AQ scores, gender, and ages of each participant**.

AQ score	Gender	Age
9	F	19
14	F	26
14	M	22
15	M	24
17	F	25
19	M	21
23	F	26
23	F	19
24	M	31
30	F	30
34	F	30

### Behavior

Mean response time (RT) to report targets in the collinear condition was 788 ms and mean RT in the orthogonal condition was 780 ms. Mean d prime was 4.6 in both the collinear and orthogonal conditions. Autism Quotient score did not correlate with RT (*R* = 0.04, *p* = 0.90) nor with d prime (*R* = 0.07, *p* = 0.83).

### fMRI

Consistent with prior reports of orientation-specific surround suppression (e.g., Blakemore and Tobin, 1972; Cavanaugh et al., 2002), a *t*-test comparing the response to the center target in the collinear vs. orthogonal conditions revealed significantly lower percent signal change in response to the target when it was surrounded by collinear (0.43) relative to orthogonal (0.61) flankers (*t*_(10)_ = −2.6446, *p* < 0.05). Figure 2B shows the average time courses for lower vs. higher AQ participants, and Figure 2C shows the mean percent signal change in each condition for each participant, showing a reduced difference between collinear and orthogonal conditions with increasing AQ score. Suppression indices ranged from −1.05 (i.e., the greatest degree of surround suppression) to 0.1 (i.e., the lowest degree of suppression), and the median index was −0.23. Figure 2D plots each participant’s SI against his/her AQ score, showing a linear relationship between the two, with greater AQ scores associated with less negative suppression indices (i.e., less surround suppression). This relationship was corroborated statistically, with a significant positive correlation between AQ score and SI (*R* = 0.76, *p* < 0.01). When considering the mean percent signal change in the collinear and orthogonal conditions separately, there was a correlation with AQ in the collinear condition (*R* = 0.70, *p* < 0.05), whereas there was no correlation with AQ in the orthogonal condition (*R* = 0.23, *p* = 0.49).

*Post-hoc* inspection of the ages of our participants also revealed a correlation between age and AQ (*R* = 0.67, *p* < 0.05), and age also correlated with SI (*R* = 0.62, *p* < 0.05). To evaluate whether AQ score accounted for more of the variance in SI than age alone, we conducted a regression analysis with SI as our independent variable and age and AQ as predictors. This analysis revealed that AQ alone accounted for 57% of the variance in SI, whereas age alone accounted for only 38% of the variance.

## Discussion

Participants attended to a target Gabor in the periphery that was flanked by Gabors that either had the same or orthogonal orientation. Mean percent BOLD signal change was measured in the V1 in each condition, and a SI was calculated that indicated the degree of surround suppression for each participant. Participants also completed the AQ questionnaire to assess the degree to which they exhibited autistic tendencies. Autism Quotient score was positively correlated with SI; higher AQ scores (higher autistic tendencies) were associated with more positive suppression indices (less surround suppression). These results are consistent with previous behavioral studies suggesting reduced surround suppression in individuals with high AQ scores (Van Heer and Crewther, 2012) and in individuals diagnosed with ASD (Foss-Feig et al., 2013). Autism Quotient score did not correlate with task performance at detecting contrast changes in the peripheral target Gabor, suggesting that the correlation with AQ was not due to a differential ability in attending to the periphery but rather due to a difference in magnitude of surround suppression as a function of AQ. Autism Quotient score correlated with the response in the collinear condition, but did not correlate with the response in the orthogonal condition, suggesting that suppression was the key factor that varied with AQ.

Age also correlated with SI, suggesting reduced surround suppression with increasing age. These results are consistent with prior studies that have suggested a reduction in surround suppression with increasing age (e.g., Betts et al., 2005; Karas and McKendrick, 2009, 2012). However, those studies compared an “older group” of individuals with mean age 68, 66, and 69, respectively, to “younger group” of individuals with mean age 23, 24, and 25, respectively. Karas and McKendrick (2012), who reported the age range of each group, indicated that the young group encompassed individuals aged 20–34. That is, all of our participants would be included in the “younger” group in these prior studies. Future research is needed to evaluate whether surround suppression also varies with increasing age in younger individuals such as the age range used in our study. Despite the correlation with age and surround suppression, a regression analysis found that AQ alone accounted for more of the variance (57%) in SI than age alone (37%), suggesting that the correlation with AQ could not be solely attributed to age.

The fact that less surround suppression was associated with higher autistic tendencies is in line with the local processing bias and/or global processing deficit seen in individuals with ASD (e.g., Shah and Frith, 1993; Jolliffe and Baron-Cohen, 1997; Plaisted et al., 1998; O’Riordan and Plaisted, 2001; O’Riordan et al., 2001; Mottron et al., 2003, 2006; O’Riordan, 2004; Kemner et al., 2008). The results from this study show a reduction in global contextual processing in individuals with high autistic tendencies as early as the V1, providing a possible neural mechanism underlying the perceptual abnormalities associated with ASD. Surround suppression has traditionally been considered to be a low-level mechanism arising from mutual inhibition of nearby neurons in V1 that have overlapping receptive fields (Gilbert and Wiesel, 1990; Adesnik et al., 2012), or from feedforward projections from the retina (Solomon et al., 2006) or thalamus (Alitto and Usrey, 2008). According to this model, surround suppression arises from the overlap in receptive fields of V1 neurons responding to the target and the flankers. Thus, surround suppression can reveal potential changes in local inhibitory processing, a hypothesis that has been put forth to explain perceptual changes in ASD (Tannan et al., 2008; Vandenbroucke et al., 2008; Kéïta et al., 2011). However, recent research has also shown that feedback mechanisms from extrastriate and higher-level areas into V1 also play a significant role in surround suppression (Jones et al., 2000; Angelucci et al., 2002; Bair et al., 2003; Angelucci and Bressloff, 2006; Nassi et al., 2013). Our study does not differentiate between low- and high-level contributions to surround suppression; future research is needed to disentangle the low- vs. high-level influences underlying the differences in surround suppression associated with the autistic trait.

Reduced surround suppression in individuals with high autistic tendencies is also consistent with an E/I imbalance underlying ASD (Rubenstein and Merzenich, 2003; Markram and Markram, 2010; Rubenstein, 2010; Vattikuti and Chow, 2010), suggesting a reduction in inhibitory mechanisms in the early visual cortex. Reduced inhibition is consistent with disproportionally low levels of GABAergic inhibition that have been shown in individuals with ASD, both in post-mortem tissue studies (Williams et al., 1980; Bauman and Kemper, 1985; Ritvo et al., 1986; Kemper and Bauman, 1992; Bailey et al., 1998; Blatt et al., 2001; Whitney et al., 2009), and in recent magnetic resonance spectroscopy (MRS) studies that found reduced resting GABA concentrations in individuals with ASD relative to NT controls (Harada et al., 2011; Gaetz et al., 2014; Rojas et al., 2014). However, these previous studies did not find differences in GABA concentrations in the visual cortex, and future research is needed to determine the relationship between surround suppression and cortical inhibition, and how it relates to ASD.

The present results examined how surround suppression relates to autistic tendencies in NT individuals. An important avenue for future research is to extend these findings to individuals diagnosed with ASD. Although the AQ test was designed to measure the extent to which NT individuals exhibit autistic tendencies, it is unknown whether a similar reduction in surround suppression will be found in individuals with ASD, given the high degree of variability in visual processing characteristics across individuals with ASD (e.g., Spiker et al., 2002; Volkmar et al., 2004; Newschaffer et al., 2007; Rice et al., 2012). Heterogeneity of ASD symptoms are found even in individuals with similar IQs, and could explain disparate findings across studies. For example, while some studies found a global processing deficit in individuals with ASD (Rinehart et al., 2000, 2001; Behrmann et al., 2006; Wang et al., 2007; Katagiri et al., 2013), other studies using similar paradigms did not find such a deficit (Ozonoff et al., 1994; Plaisted et al., 1999; Iarocci et al., 2006; Hayward et al., 2012). An interesting question is whether the degree to which a given individual exhibits a local processing bias and/or global processing deficit relates to the degree to which he/she exhibits surround suppression. The results from the present study suggest a difference in surround suppression mechanisms in early visual cortex of individuals with high autistic tendencies, providing a starting point for future work examining the neural substrates of perceptual processing differences found in individuals with ASD.

## Conflict of interest statement

The authors declare that the research was conducted in the absence of any commercial or financial relationships that could be construed as a potential conflict of interest.
